# Characterizing the impact of spatial clustering of susceptibility for measles elimination

**DOI:** 10.1016/j.vaccine.2018.12.012

**Published:** 2019-01-29

**Authors:** Shaun A. Truelove, Matthew Graham, William J. Moss, C. Jessica E. Metcalf, Matthew J. Ferrari, Justin Lessler

**Affiliations:** a Department of Epidemiology, Johns Hopkins Bloomberg School of Public Health, Johns Hopkins University, Baltimore, MD, USA; b The Hospital for Tropical Diseases, Wellcome Trust Overseas Programme, Oxford University Clinical Research Unit, Ho Chi Minh City, Viet Nam; c Department of Ecology and Evolutionary Biology, Princeton University, Princeton, NJ, USA; d Office of Population Research, Woodrow Wilson School, Princeton University, Princeton, NJ, USA; e Center for Infectious Disease Dynamics, Pennsylvania State University, University Park, PA, USA; f Department of Biology, The Pennsylvania State University, University Park, PA, USA

**Keywords:** Heterogeneity, Clustering, Measles, Effective reproductive number, Critical vaccination threshold, Critical immunity threshold, Disease transmission, Outbreak risk, Vaccination, Stochastic individual-based model

## Abstract

Measles elimination efforts are primarily focused on achieving and maintaining national vaccination coverage goals, based on estimates of the critical vaccination threshold (*V_c_*): the proportion of the population that must be immune to prevent sustained epidemics. Traditionally, *V_c_* estimates assume evenly mixing populations, an invalid assumption. If susceptible individuals preferentially contact one another, communities may remain vulnerable to epidemics even when vaccination coverage targets are met at the national level. Here we present a simple method to estimate *V_c_* and the effective reproductive number, *R*, while accounting for spatial clustering of susceptibility. For measles, assuming *R_0_* = 15 and 95% population immunity, adjustment for high clustering of susceptibility increases *R* from 0.75 to 1.29, *V_c_* from 93% to 96%, and outbreak probability after a single introduction from <1% to 23%. The impact of clustering remains minimal until vaccination coverage nears elimination levels. We illustrate our approach using Demographic and Health Survey data from Tanzania and show how non-vaccination clustering potentially contributed to continued endemic transmission of measles virus during the last two decades. Our approach demonstrates why high national vaccination coverage sometimes fails to achieve measles elimination, and that a shift from national to subnational focus is needed as countries approach elimination.

## Highlights

• Outbreak potential is greater than expected if non-vaccination is clustered.
• Vaccination targets are insufficient to achieve herd immunity in many settings.
• Impact of susceptibility clustering highest in countries near disease elimination.
• Countries with high vaccination should shift focus to local vaccination targets.

## 1 Introduction

Despite significant improvements in global measles vaccination coverage (from 73% to 85% for a single dose between 2000 and 2015), large outbreaks continue to occur, making declared goals of measles elimination by 2020 across World Health Organization (WHO) regions realistically unattainable [1], [2]. These outbreaks have occurred both in countries with low measles vaccination coverage and those with perceived high levels of measles control throughout Europe (e.g., France, Romania), Africa (e.g., Malawi), and Asia (e.g., the Philippines) [3]. The continued occurrence of outbreaks reflects the considerable logistical, political, and social challenges to measles elimination, highlighting the need for novel approaches [4], [5].

WHO’s vaccination coverage goals of 95% for the first and second doses of measles-containing vaccine (MCV) in the Global Measles and Rubella Strategic Plan derive, in part, from theoretical estimates of the immunity level needed to prevent ongoing measles virus transmission [1], [6]. These estimates are based on the basic reproductive number (*R_0_*), the average number of people a single infected person is expected to infect in a fully susceptible population (usually assumed to be 12 to 18 for measles but varies substantially) [7], [8], [9]. Theory states that if a country can successfully immunize, via vaccination, a proportion of their population equal to 1 − (1/*R_0_*), the population will have sufficient herd immunity to avoid significant epidemics if introduction of the pathogen occurs [7], [10]. Hence, for measles, 92–94% of the population would need to be immune to achieve this critical vaccination threshold ($V_{c}$).

Though useful, this rule of thumb is based on simple epidemic models that ignore population structure, assuming each infected individual is equally likely to have a potentially infectious contact with every other individual in the population. If this assumption is violated, then predictions from these models may be invalid. Specifically, in a setting where susceptible individuals are more likely to be in contact with other susceptible individuals than would be expected by random chance, an introduced pathogen has a higher probability of producing uninterrupted chains of transmission than in a setting where contact is homogeneous. If vaccination coverage is geographically or socially heterogeneous, the vaccination process itself can lead to violation of this critical assumption [11], [12], [13].

The existence of heterogeneities in vaccination coverage and their association with disease incidence has been repeatedly demonstrated. Within African countries, heterogeneity in measles vaccination coverage is prevalent, with clusters of low vaccination common even in countries with high overall coverage [14]. In the United States, spatial clustering of nonmedical exemptions to school immunization requirements in Michigan and California were associated with pertussis incidence [15], [16]. In 2014, an outbreak in Ohio of 380 confirmed measles cases occurred in a large Amish community [17]. The spatial clustering of this population, where vaccination coverage may be as low as 8%, resulted in the largest measles outbreak in recent U.S. history, despite high measles vaccination coverage for Ohio overall (>92% among kindergarteners) [18].

The impact of these heterogeneities on disease transmission is well supported by theoretical work. Recent work by Funk et al*.* demonstrated the increased importance of immunity in specific age groups, particularly 5–9 year-olds, due to age-specific heterogeneity in contact patterns [19]. Other studies demonstrated that assuming homogeneous contact in transmission networks can underestimate $R_{0}$ [10], [20], [21]. Using network theory, Salathe and Bonhoeffer found that when heterogeneity in contact between susceptibles exists, a higher level of vaccination coverage is needed to eliminate disease transmission than estimated from homogeneous networks [21].

Simple, broadly applicable, evidence-based methods that provide guidance where heterogeneity in vaccine use exists may be useful in planning and evaluating measles control activities. Here we present a method for estimating the effective reproductive number and critical vaccination threshold in the presence of spatial heterogeneity in susceptibility. Using Demographic and Health Survey (DHS) data from Tanzania, we demonstrate its application and examine how spatial heterogeneity in vaccination coverage may have contributed to continued measles virus transmission while vaccination coverage, measured at the national level, was presumed to be high enough to achieve elimination.

## 2 Methods

### 2.1 Approach

The basic and effective reproductive numbers, *R_0_* and *R*, are key to estimating disease outbreak potential and the vaccination coverage needed to interrupt transmission. *R* is how many people an average infectious individual is expected to infect in a population with some immunity. If everyone is equally likely to interact with everyone else, the probability that a contact is susceptible equals the proportion susceptible in the population. When immunity is exclusively vaccination-derived, this proportion susceptible is *1-v*, where *v* is the proportion who successfully develop protective immunity following vaccination. Hence:

(1) $$R=R_{0}\times \mathrm{Pr}\left(contact\;is\;susceptible\right)=R_{0}\left(1-v\right)$$

Here we focus on maintaining measles elimination through successful vaccination. For simplicity, we ignore the impact of immunity acquired through infection, assuming application to populations where endemic measles has been eliminated (i.e., where we assume susceptibility is synonymous with not being successfully vaccinated).

In many populations spatial heterogeneity in vaccination arises from access or refusal, and individuals are, in general, more likely to contact people living near their home than those living farther away [22], [23], [24], [25]. The more spatially clustered non-vaccination is, the higher the probability that contacts of a susceptible individual are also susceptible, and the higher the probability of onward transmission if a pathogen is introduced.

We can account for this heterogeneity in Eq. (1) by adjusting the probability that a contact is susceptible. We quantify the tendency of non-vaccination to cluster using the statistic $\tau \left(r\right)$, defined as the relative probability that an individual living at spatial distance r from an unvaccinated individual is also unvaccinated versus the probability that anyone in the population is unvaccinated [26], [27]. Hence:

(2) $$\mathrm{Pr}\left(contact\;is\;susceptible\right|contact\;lives\;at\;distance\;r)=\left(1-v\right)\frac{1-v\left(r\right)}{1-v\left(\infty \right)}=\left(1-v\right)\tau \left(r\right)$$

where $1-v\left(\infty \right)$ is equivalent to 1-v for the full population. If we know $g\left(r\right)$, the probability that a contact occurs at distance *r* given that a contact occurred, we can calculate the number of infectious contacts that a susceptible individual is expected to make (i.e., the adjusted effective reproductive number, $R^{*}$):

(3) $$R^{*}=\beta c\left(1-v\right)\int_{0}^{\infty }g\left(r\right)\tau \left(r\right)dr=R_{0}\left(1-v\right)\int_{0}^{\infty }g\left(r\right)\tau \left(r\right)dr=R_{0}\left(1-v\right)\phi$$

where *c* is an individual’s expected number of potentially infectious contacts over the course of their infectious period, β is the per contact transmission probability (note that $R_{0}=\beta c$), and ϕ is a clustering adjustment factor for R (i.e., $R^{*}=R\phi$).

The critical vaccination threshold, $V_{c}$, is calculated as the vaccination level where *R* < 1, or $V_{c}=1-\frac{1}{R_{0}}$. Assuming the susceptibility clustering and the distribution of contacts remain stable, we can account for clustering in an adjusted critical vaccination threshold, $V_{c}^{*}$, by including ϕ:

(4) $$V_{c}^{*}=1-\frac{1}{R_{0}\phi }$$

## 3 Statistical methods

For these analyses, we assumed that the relative probability that an individual at distance r from an unvaccinated (i.e., susceptible) individual is also unvaccinated follows an exponential decay function, of the form $\tau \left(r\right)=\theta e^{\left(-\lambda r\right)}+1$, where θ is the maximum relative probability of a contact being unvaccinated and λ is the rate of decay towards 1. In simulations, we assume successful vaccination to be the only source of immunity in the population. The distance from the home at which contacts occur is assumed to follow a gamma distribution, $g\left(r\right)\;gamma\left(r,\alpha ,\beta \right)$, where r is the distance of the contact. We note that the method could be applied using other parametric and empirical forms of $\tau \left(r\right)$ and $g\left(r\right)$.

## 4 Analytic examples and simulation studies

To illustrate our approach, we considered populations with four levels of spatial clustering of susceptibility: *none* ($\tau \left(r\right)$ parameters: θ = 0, λ = 0.5), *low* (θ = 0.25, λ = 0.5), *moderate* (θ = 0.5, λ = 0.5), and *high* (θ = 1.0, λ = 0.5). We examined contact distributions fit to empirical data from three distinct populations representing different contact patterns: $g_{A}\left(r\right)$, fit to data from urban and rural locations in southern China (α = 0.238, β = 0.162); $g_{B}\left(r\right)$, fit to data from rural Zambia (α = 0.086, β = 0.042); and $g_{C}\left(r\right)$, fit to cell phone data from the U.S (α = 0.701, β = 0.076) (see Supplemental Figure S1 and Text S4 for full details) [28], [29], [30]. Contacts are most local in $g_{A}\left(r\right)$, while they are most dispersed in $g_{C}\left(r\right)$, reflecting US commuting culture. We considered three levels of successful vaccination coverage, 85, 90, and 95%, and assumed *R_0_* = 15 (similar to measles).

**Fig. 1:**
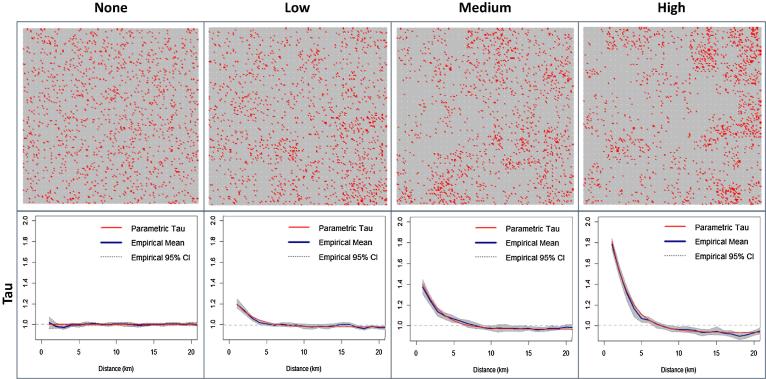
Synthetic populations and corresponding *τ(r)* functions with *no*, *low*, *medium*, and *high* spatial clustering of susceptibility. Individuals are spatially distributed evenly across the space, and susceptibility increasingly clustered (grey = immune, red = susceptible).

To validate analytic calculations, we performed spatially explicit simulation studies of these scenarios using stochastically generated populations of 100,000 individuals for each level of vaccination coverage and clustering. We performed sensitivity analyses of the impact of population size and density (Fig. 1, Appendix S7). Assuming a single introduction of a measles-like virus (*R_0_* = 15, serial interval of 2 weeks), we performed 10,000 simulations for each scenario. $R^{*}$ was calculated analytically for each scenario using the pre-defined target $\tau \left(r\right)$ and the exact $g\left(r\right)$ from each simulation. For each scenario we compared the behavior (outbreak probability and final size distribution) of the spatially explicit model with that of a homogenous mixing model where $R_{0}$ was equal to the corresponding $R^{*}$. An outbreak was defined as ≥5% of susceptible individuals becoming infected.

## 5 Example application

We illustrate our approach using data from Demographic and Health Surveys (DHS) conducted in Tanzania. DHS are cluster randomized surveys that include reported and documented individual-based vaccination histories, and, in the most recent surveys, GPS coordinates for sampling cluster locations [31]. These data provide a snapshot of vaccination coverage achieved through routine and supplemental immunization activities among children under 5 years. We use Tanzania to demonstrate the application of our approach because the country had multiple years of DHS surveys with spatial data and a recent history of increasing measles vaccination coverage. See Supplemental Text S9 for full methods.

All analyses were performed using R 3.4.1 and RStudio 1.0.153.

## 6 Results

### 6.1 Analytic Analysis

Analytic results show that clustering of non-vaccination can have a significant impact on both R and $V_{c}$ (Table 1 and S1). Under contact distribution $g_{A}\left(r\right)$ and *high* clustering, R* is nearly double that assumed by standard estimates that assume homogenous mixing (ϕ = 1.71), and $R^{*}$ > 1 at 95% successful vaccination coverage (i.e., herd immunity is not achieved, Table 1). These effects translate into significant increases in $V_{c}^{*},$ from 93% to 96% (Table 1).

**Table 1:** Analytic $R^{*}$ and $V_{c}^{*}$ estimates from Eq. (1) assuming $R_{0}$ = 15 and using contact distributions derived from Read et al. 2014 (*g_A_(r)*), Searle et al. 2017 (*g_B_(r)*), and Noulas et al. 2012 (*g_C_(r)*) [28], [29], [30].

Vaccination coverage	Clustering	*g_A_(r)*		*g_B_(r)*		*g_C_(r)*	
		R*†	V_c_*	R*†	V_c_*	R*†	V_c_ *
95%	*None*	0.75	93.3%	0.75	93.3%	0.75	93.3%
	*Low*	0.88	94.3%	0.90	94.4%	0.80	93.7%
	*Medium*	1.02	95.1%	1.05	95.2%	0.84	94.0%
	*High*	1.29	96.1%	1.35	96.3%	0.93	94.6%

† Assumes R_0_ = 15 and λ = 0.5.

**Fig. 2:**
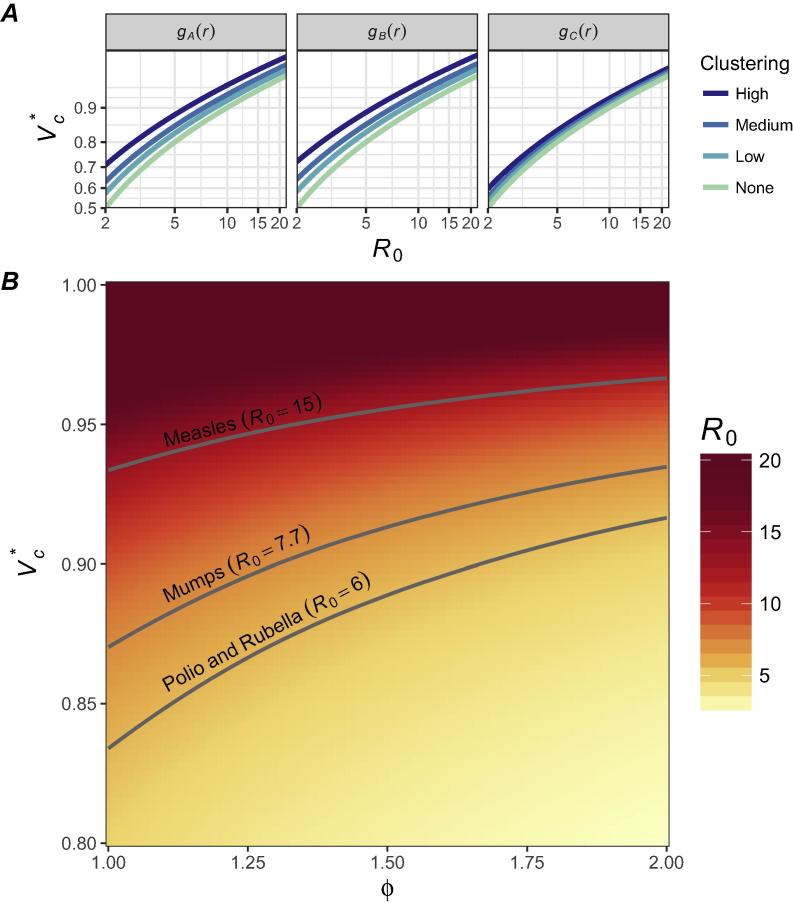
The association between $R_{0}$, $V_{c}^{*}$, and spatial clustering of non-vaccination (ϕ). As clustering increases, the required vaccination coverage to maintain R = 1 increases. At low $R_{0}$, this increase due to clustering is much greater than at high $R_{0}$. (A) The association between $R_{0}$ and $V_{c}^{*}$ at the four defined levels of clustering, using the three $g\left(r\right)$ distributions. The relative increase in $V_{c}$ with increased clustering is relatively equivalent for $g_{A}\left(r\right)$ and $g_{B}\left(r\right)$, but lower for $g_{C}\left(r\right)$. (B) The relationship between $V_{c}^{*}$, ϕ, and $R_{0}$, where ϕ represents the relative impact of spatial clustering on $R^{*}$. This relationship is highlighted for measles ($R_{0}$ = 15), mumps ($R_{0}$ = 7.7), and polio and rubella ($R_{0}$ = 6) [10], [32], [34], [33]. For diseases like polio, with lower $R_{0}$, there is a substantially greater increase in absolute $V_{c}^{*}$ with increasing ϕ, as compared to diseases with higher $R_{0}$.

The absolute increase in $V_{c}^{*}$ due to clustering is inversely related to $R_{0}$ (Fig. 2). For diseases with high $R_{0}$ like measles ($R_{0}$ = 15), the required $V_{c}^{*}$ with *high* clustering is 3% higher than without (Table 1). Diseases with potentially lower $R_{0}$ like mumps ($R_{0}$ = 7.7), polio ($R_{0}$ = 6), rubella ($R_{0}$ = 6) or cholera ($R_{0}$ = 2.6) have larger, yet possibly more manageable, increases in $V_{c}^{*}$, of 6% (87% versus 93%), 8% (83% versus 91%), and 19% (62% versus 81%) [32], [10], [33], [34], [35].

**Fig. 3:**
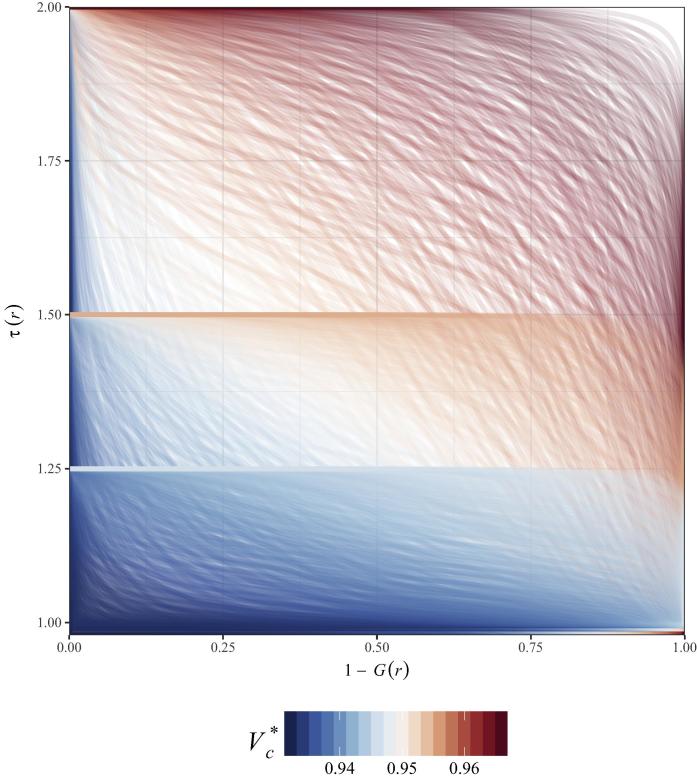
Critical vaccination threshold, as defined by the overlapping densities of the $\tau \left(r\right)$ and $g\left(r\right)$ distributions. Plotted curves are $\tau \left(r\right)$ where r is that which corresponds to the value of $1-G\left(r\right)$, and color corresponds to the resulting $V_{c}^{*}$ when $R_{0}$ = 15. Three levels of clustering (θ = 0.25, 0.50, 1.0) and 529 $g\left(r\right)$ distributions, with both α and β range = 0.1–10.

The shapes of both the contact distance and clustering distributions affect the resulting estimates of $R^{*}$, $V_{c}^{*}$, and ϕ. Of the three contact distributions applied, $g_{B}\left(r\right)$ produced the highest $R^{*}$ values in the presence of clustering due to having the highest proportion of contacts within short distances; the more dispersed $g_{C}\left(r\right)$ produced the lowest $R^{*}$ (Table 1). Curves of $1-G\left(r\right)$ (where G(r) is the cumulative distribution function of $g\left(r\right)$) versus $\tau \left(r\right)$ demonstrate the effect of overlap between $\tau \left(r\right)$ and $g\left(r\right)$ (Fig. 3). The impact of clustering is maximized with perfect overlap between the $g\left(r\right)$ probability and maximum $\tau \left(r\right)$ value, occurring when 100% of contact between individuals occurs within the distance where $\tau \left(r\right)$ is highest, in this case at r = 0. The impact of clustering is reduced when clustering and contact occur at different distances. Theoretically, if the shapes of the $\tau \left(r\right)$ and $g\left(r\right)$ functions were mismatched, we could see $V_{c}^{*}$ < $V_{c}$. However, for this study we constrained these functions to monotonically decreasing distributions, thus $V_{c}^{*}$ approaches $V_{c}$ as $g\left(r\right)$ approaches a uniform distribution (i.e. equal contact distance probability, or homogeneous contact).

## 7 Simulation studies

We found near perfect correlation between the expected outbreak probabilities from homogeneous mixing SIR simulations and spatial mixing simulations (Pearson’s ρ = 0.98) and high comparability between these estimates (Pearson’s $\chi^{2}$ *p* = 0.24) (Table 2). However, the final sizes of these epidemics differed significantly (51% [95% CI: 42–60%] vs 82% [95% CI: 80–84%] at $R^{*}$ = 2.0), due to previously described differences between spatial and homogenous mixing populations [36].

**Table 2:** Outbreak probability from simulations with SIR and individual-based spatially explicit model, assuming λ = 0.5 and *g(r)*.

Vaccination coverage	Clustering	R*‡	Probability of outbreak†	
			Spatial simulations, mean (95% CI)	SIR simulations, mean (95% CI)
95%	*None*	0.75	0 (0–0.01)	0 (0–0)
	*Low*	0.88	0.01 (0.00–0.02)	0.01 (0.01–0.01)
	*Medium*	1.02	0.07 (0.06–0.08)	0.07 (0.07–0.08)
	*High*	1.24	0.23 (0.22–0.24)	0.23 (0.22–0.24)

† Outbreak is defined by ≥5% of the susceptible individuals becoming infected. Assumes introduction happens at equal rates randomly among susceptibles.

‡ $R^{*}$ calculated analytically using the empirical g(r) from spatial simulations and defined τ(r) distributions.

**Fig. 4:**
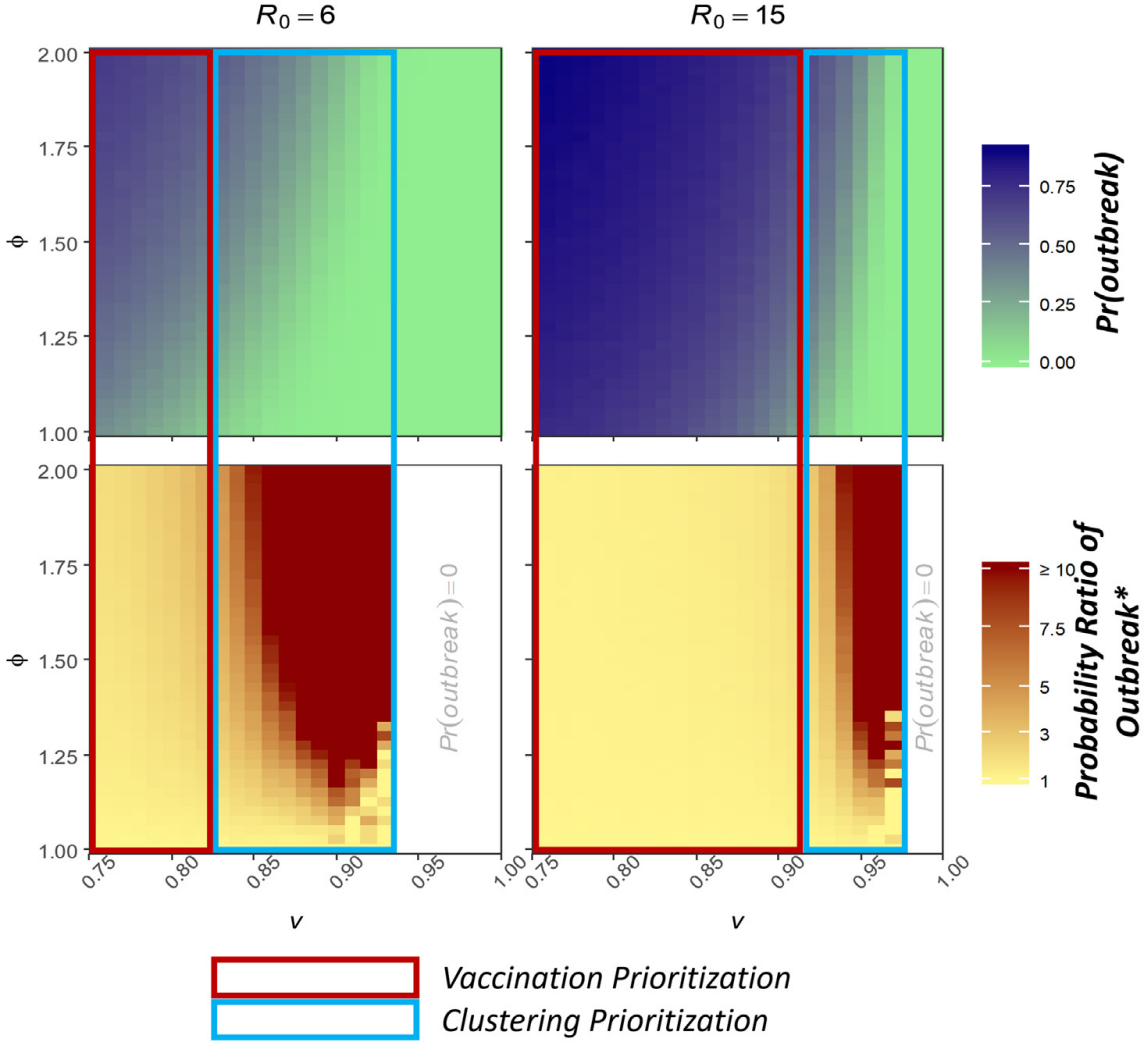
Probability of an outbreak and the outbreak probability ratio given adjustment for clustering (ϕ) compared with homogeneous (ϕ = 1), for $R_{0}$ = 6 and $R_{0}$ = 15. The probability ratio compares the clustering-adjusted estimates of outbreak probability for each level of clustering to the homogeneous estimates at each level of successful vaccination coverage. The probability of an outbreak decreases with increasing successful vaccination proportion (*v*) and increases with increasing clustering of susceptibility (ϕ). Until vaccination becomes high enough, the contribution of clustering is negligible and public health focus should be on increasing vaccination coverage, as highlighted by the areas in the red boxes. As successful vaccination coverage approaches elimination levels (estimated at 83% for $R_{0}$ = 6 and 93% for $R_{0}$ = 15 with the traditional critical immunization threshold equation) the relative effect of clustering on the outbreak probability increases exponentially. When vaccination is sufficiently high, prioritization should shift from increasing national vaccination coverage to the clustering of susceptibility (blue box).

The relative impact of non-vaccination clustering on outbreak probability increases exponentially as vaccination coverage approaches the critical vaccination threshold (i.e., near elimination; Fig. 4). For measles, the ratio of the outbreak probability assuming high clustering to the outbreak probability assuming no clustering (hereafter PrR), with 85% immunity was 1.4 (95% CI: 1.3–1.5) and with 90% immunity PrR = 1.9 (95% CI: 1.7–2.1), increasing rapidly to PrR > 20 with 95% immunity (Pr(outbreak) = 23% [95% CI: 22–24%] vs. 0% [95% CI: 0–0.1%]) (Table 3 and S3).

**Table 3:** Incidence and estimated effective reproductive numbers for Tanzania from 1998 to 2014.

Year	5-yr routine vaccination	5-yr incidence (per 100,000)	*R,* 5-year mean		*V_c_*	
			*Unclustered*	*Clustered*†	*Unclustered*	*Clustered*†
1999	76.2%	29.78	3.57	4.5 (3.9–5.3)	93.3%	94.7% (93.9–95.5%)
2010	92.2%	3.75	1.17	1.8 (1.5–2.3)	93.3%	95.7% (94.7–96.5%)

† Estimate mean and 95% CI.

## 8 Application

**Fig. 5:**
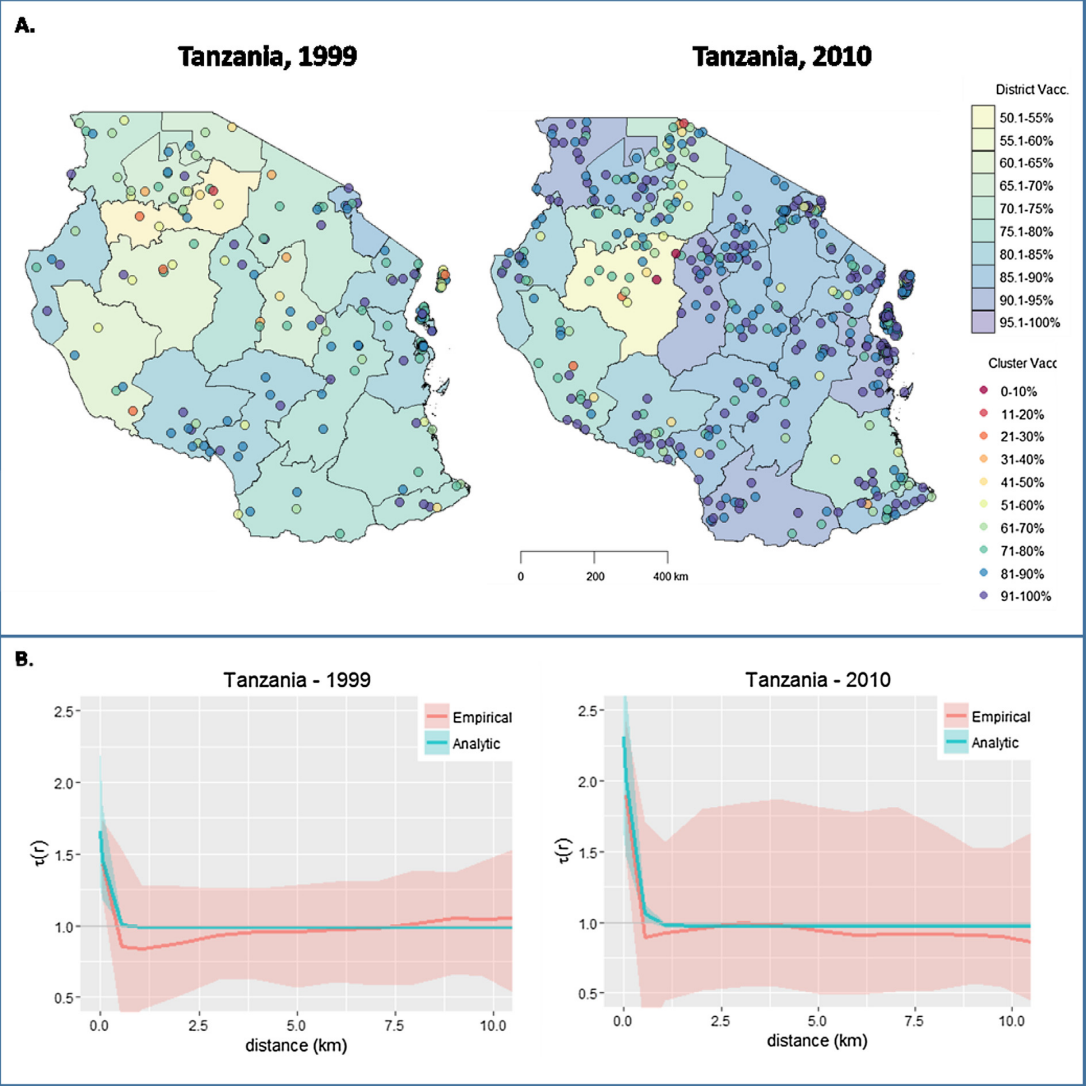
(A) Vaccination coverage among children aged 1–5 years by district and survey sample in Tanzania during the 1999 and 2010 Demographics and Health Surveys. (B) Empirical *τ(r)* functions and fitted exponential for Tanzania and Zambia, using DHS cluster data.

We illustrate our approach using DHS data from Tanzania, which has a recent history of largely uninterrupted measles virus transmission concurrent with increasing vaccination coverage. Visual examination of cluster and district-level vaccination coverage shows apparent clustering of non-vaccination during both the 1999 and 2010 Tanzania DHS surveys, with a possible increase in clustering in 2010 compared to 1999 (Fig. 5a). Fitting $\tau \left(r\right)$ to DHS data confirms this observation, with significant clustering of non-vaccination in Tanzania during the 1999 and 2010 surveys, with ϕ = 1.26 (95% CI: 1.1–1.5) and 1.56 (95% CI: 1.2–1.9) (Fig. 5b). Assuming stable clustering and applying these ϕs to 5-year means of UNICEF/WHO vaccination coverage, R was likely substantially higher than implied by observed coverage in Tanzania. In 1999, with v = 76% and assuming $R_{0}$ = 15, $R^{*}$=4.50 (95% CI: 3.90–5.30) versus *R* = 3.57; in 2010, withv = 92%, $R^{*}$=1.83 (95% CI: 1.46–2.25) versus *R* = 1.17 (Table 3) [37]. Starting in 2012, $R^{*}$<1 and vaccination > $V_{c}^{*}$, assuming accurate vaccination coverages, and this was followed by measles incidence dropping and remaining below 1 per 100,000 (Table S2). Without reductions in clustering of non-vaccination, Tanzania will likely need to maintain successful vaccination coverage of at least 95–96% to maintain interrupted measles virus transmission as naturally-acquired immunity wanes.

## 9 Discussion

We demonstrate that the assumption of homogeneity under which standard methods function likely results in the underestimation of the effective reproductive number and critical immunity threshold, especially where high clustering of susceptibility exists. This can lead to situations where infectious diseases continue to spread despite perceived control and elimination. The effective reproductive number increases with increasing susceptibility clustering and the impact of this clustering on the relative probability of an outbreak becomes exponentially higher as countries approach elimination levels of vaccination (e.g., 95% for measles). Global MCV1 coverage currently stands at 85%, with 40% of 194 countries meeting the 95% MCV1 coverage target, putting these countries and others striving to meet it in the critical range where effective epidemic risk assessment and vaccination strategy prioritization now require accounting for clustering [38].

Our results can help guide prioritization between overall vaccination coverage and reducing clustering of non-vaccination (Fig. 4). Where national measles vaccination coverage remains well below targets (i.e. ≤ 90%), efforts to increase overall vaccination coverage likely provide substantially more benefit than efforts to reduce or address heterogeneities in vaccination coverage. However, where national vaccination coverage is near classical herd immunity levels, non-vaccination clustering has a strong impact on transmission potential; thus, strategies to account for and address this clustering may be imperative to elimination success.

As demonstrated with our derivation of an adjusted critical immunity threshold equation (Eq. (4)), one theoretical strategy to counter susceptibility clustering may be increased national vaccination targets. This may be useful where clustering is relatively low, as $V_{c}^{*}$ ≤ 95% for measles with *low* or *medium* clustering. Thus, assuming successful immunization, current 95% targets may be sufficient to counter the effects of clustering [1]. However, where 95% coverage is unlikely or clustering is high, alternative strategies to address clustering directly are likely necessary.

Country-specific strategies, such as targeting specific vaccine deployment or individual characteristics might be effective. For example, clustering of non-vaccination has been associated with distance from health care and lack of health education, as well as geographic isolation and belonging to nomadic groups [39], [40]. The challenge of these strategies is that they rely on knowledge of the specific causes of heterogeneity across a population. A more effective universal approach may be to shift from national targets once vaccination coverage reaches a threshold, such as 90% for measles, to local (i.e., regional, provincial) vaccination targets, and following from these local targets, local vaccination activities.

Though developed using measles, application of these methods readily extends to other infectious diseases, including pertussis, mumps, rubella, and polio, all of which currently present challenges worldwide. For example, recent mumps outbreaks among college students and hockey teams may be the result of clustering of susceptibility due not to non-vaccination but to immunity waning, stemming from clustering by age [41]. With $R_{0}$ = 7.7, booster vaccination coverage of 93% among the student populations should be highly successful at producing herd immunity despite high susceptibility clustering. For diseases with lower *R_0_* values, like mumps, our methods may more readily translate into feasible adjusted overall vaccination targets.

To provide a useful general rule of thumb for vaccination, we made several simplifying assumptions, in particular focusing on immunity acquired through successful vaccination. As such, our model did not explicitly account for naturally-acquired immunity, vaccine efficacy (VE), or multiple doses of vaccine, though it is possible to incorporate these effects, given available data (Supplemental Text 10.2).

Naturally-acquired immunity presents challenges to accurately estimating $R^{*}$, and if it occurs in a spatially localized manner, such as through an outbreak in a community, the nature of susceptibility clustering may change. However, as noted above, the greatest impact of clustering, and where this framework is most valuable, is when populations are close to disease elimination. While naturally-acquired immunity likely provides protection that our model does not account for, without continued transmission in these populations susceptibility will eventually mirror effective vaccination. For our example country Tanzania, because of existing natural immunity, our $R^{*}$ estimates may be inflated. However, to sustain the recent successful interruption of transmission in Tanzania, immunization programs will likely need to maintain vaccination coverage higher than 95%, especially as the proportion of the population with natural immunity declines. Use of serological surveys to detail susceptibility (rather than vaccination coverage surveys) could circumvent the challenge posed by natural immunity, as serological surveys have the additional benefit of capturing clustering of naturally-acquired immunity [42], [43].

Describing human contact introduces further challenges. We simplified contact into a spatial proximity construct defined by a gamma probability model, a simplistic approximation that we acknowledge does not fully capture the complexity of human social networks and mixing. Future explorations of the effects of susceptibility clustering will likely require more complex models of social interaction and behavior. Among these, age-assortativity, which has been well-described by the POLYMOD study, has previously been shown by Funk et al. to produce a similar effect to spatial clustering if susceptibility is age-clustered [44], [19]. Other assortativity research, such as that defined by the Social Identity Theory and Self-Categorization Theory, suggests contact is also clustered in perceived social categories or identities, in which behaviors and perceptions, including vaccination skepticism, also cluster [45]. Building on work in behavioral modeling and social psychology, we may be able to quantify this social clustering, providing a better understanding of both transmission risk, and how to specifically target these socially-defined non-vaccination clusters and prevent outbreaks. This may be especially important as populations near elimination. Research on behavioral modeling and game theory suggests that, as people perceive less risk from the disease due to lower prevalence, their incentive to vaccinate may also decrease [46]. This may already be true in the U.S. and Europe [47].

Acquiring high-quality and sufficient data is critical to approximating susceptibility clustering and human contact patterns. For spatially-defined non-vaccination clustering and contact, we were able to leverage widely available DHS data, with which accurate estimation of spatial susceptibility clustering is likely possible without collecting new data. In settings where clustering of non-vaccination and contact patterns are less spatially-structured, available data, such as mobile phone, social networks, and school-based data, may be useful. Ideally, as recent reviews have concluded, human behavior presented within infectious disease models should be based on detailed behavioral data, particularly that of actual behavior and not just intention, and should be specific to the target population and disease [48], [49].

Even minimal clustering of non-vaccination, and resultant susceptibility, can produce substantial increases in outbreak risk, particularly for populations that are close to elimination targets. Through simple adjustments to the current rules of thumb guiding vaccination policy, we can estimate the impact of this heterogeneity and adjust strategies accordingly. For countries that are well-below current vaccination targets, the strategy should remain the same: continued efforts to achieve national vaccination coverage targets. For those near elimination, strategies that account for, or directly address, clustering of non-vaccination may yield more dividends. The approach presented here can serve to guide appropriate decision making and target setting.

## 10 Policy implications

**Figure:**
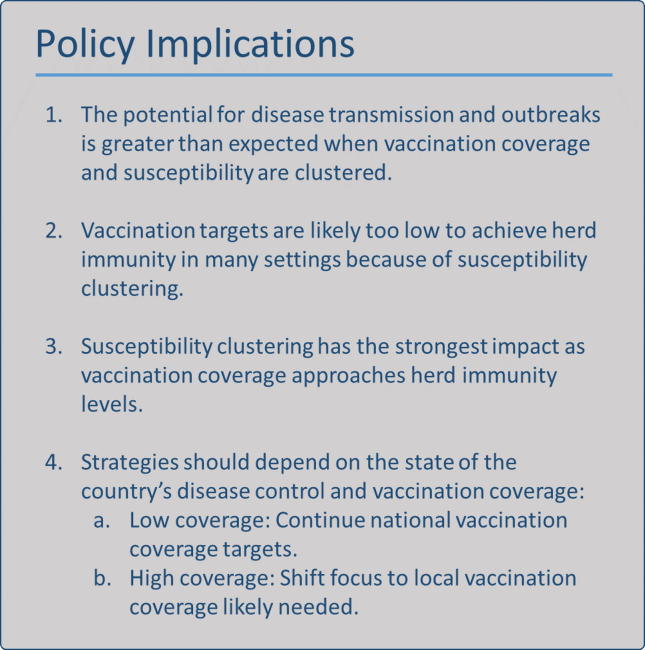

## Ethics

This research did not require ethical approval as it used publicly available de-identified data (DHS).

## Competing interests

We have no competing interests.

## Authors' contributions

Conception and design of study: SAT and JL.

Acquisition of data: SAT.

Development and/or verification of analytic methods: SAT, JL, MG, CJEM.

Analysis and/or interpretation of data: SAT, JL, WJM.

Drafting the manuscript: SAT and JL.

Revising the manuscript: SAT, JL, MG, WJM, CJEM, MJF.

Approval of the final manuscript: SAT, JL, MG, WJM, CJEM, MJF.
