# Supplementary material for: Characterizing the impact of spatial clustering of susceptibility for measles elimination
Source: Vaccine. 2019 Jan 29;37(5):732–41. doi: 10.1016/j.vaccine.2018.12.012 (PMC6348711; doi:10.1016/j.vaccine.2018.12.012)
Supplement: Supplementary data 1 [file mmc1.docx]

**Supplement to Characterizing the impact of spatial clustering of susceptibility for measles elimination**

**SUPPORTING INFORMATION**

**Truelove et al.**

# Derivation of Spatially-Structured Effective Reproductive Number Estimate

The standard equation for $R$ effective, assuming homogeneous mixing:

$R=R_{0}\left( 1-v \right)$

where $v$ is the overall proportion vaccinated in the population. We assume that $v$ is equal to the proportion *successfully* vaccinated, and to account for a vaccine efficacy below 100%, $v$ is simply adjusted (i.e. proportion vaccinated x vaccine efficacy). To calculate an $R$that accounts for the increased probability of contact between susceptible individuals due to spatial clustering, we modify the standard $R$ to include what is essentially a measure of the relative risk of contact between susceptible individuals due to clustering as compared with an unclustered, homogenous population. The derivation is as follows:

$R_{0}$ assumes that every contact is susceptible, or:

$$\Pr\left( susceptible=1 \right)=\frac{N_{suscept}}{N_{total}}=1$$

For a population with some level of immunity, $\Pr\left( susceptible contact \right)\neq1$ for the full population. Therefore, we calculate *R as*:

$$R=R_{0}*\Pr\left( susceptible=1 \right)$$

Under the assumption of homogeneous contact probability:

$$\Pr\left( susceptible=1 \right)=1-\Pr(susceptible=0)=1-\frac{N_{vaccinated}}{N_{total}}=1-v$$

thus we arrive at our traditional calculation for $R_{eff}$:

$$R=R_{0}(1-v)$$

To incorporate heterogeneity, we can modify this equation by modifying the probability of a contact being susceptible. First, we can define for each distance $x$ from another susceptible individual:

$$\Pr\left( susceptible=1 \cap X=x \right)=\Pr\left( X=x \right)\Pr\left( susceptible=1 \right| X=x),$$

so for all distances (the whole population):

$$\Pr\left( susceptible=1 \right)=\int\Pr\left( X=x \right)\Pr\left( susceptible=1 \right| X=x) dx$$

To quantify spatial clustering, we use the $\tau$ statistic:

$$\tau\left( x \right)=\frac{1-v\left( x \right)}{1-v\left( \infty\right)}=\frac{\Pr\left( susceptible=1 \right|X=x)}{\Pr\left( susceptible=1 \right|X=\infty)}$$

Using $g\left( x \right)$ to represent $\Pr\left( X=x \right)$, we incorporate $\tau(x)$ and$g(x)$ and get:

$$\Pr\left( susceptible=1 \right)=\int\Pr\left( X=x \right)\Pr(susceptible=1| X=x) dx$$

$$=\int g\left( x \right)*(\tau\left( x \right)*Pr(susceptible=1| X=\infty)) dx$$

$$=\Pr\left( susceptible=1 \right|X=\infty)*\int g\left( x \right)\tau\left( x \right) dx$$

Since $\Pr\left( susceptible=1 \right|X=\infty)=1-v\left( \infty\right)\approx1-v$,

$$\Pr\left( susceptible=1 \right)=(1-v)\int g\left( x \right)\tau\left( x \right) dx$$

Finally, incorporating this we get our final model:

$$R=R_{0}*\Pr\left( susceptible=1 \right)$$

$$R_{eff}=R_{0}(1-v)\int g\left( x \right)\tau\left( x \right) dx$$

# Weighted Cluster Survey $\boldsymbol{\tau}\left( \boldsymbol{r} \right)$ Calculation

To calculate an accurate $\tau(r)$ using clustered survey data with sampling weights for the clusters, we use a revised calculation to account for the structure of the data and sampling weights.

The standard equation for $\tau(r)$ is:

$$\tau\left( r_{1},r_{2} \right)=\frac{\Pr\left( z_{i}=z_{j} \right|j\in\Omega_{i}(r_{1}, r_{2}))}{\Pr\left( z_{i}=z_{j} \right|j\in\Omega_{i}(.))}$$

See Salje et al. 2014 and Lessler et al. 2016 for more details [1,2].

We designed these methods for use with both clustered survey and discrete data. As such, to use with DHS data, we aggregated the individual-level DHS data to cluster-level data, with cluster ID, geospatial coordinates, number of individuals, sampling weights, and proportion susceptible for each cluster. These methods can be directly applied to discrete data such as counties or districts.

First, we define the weighted probability of a susceptible individual within a distance range ${(r}_{1},r_{2})$ from cluster $i$:

$$\pi_{i}\left( r_{1},r_{2} \right)=\frac{\sum_{j\in\Omega_{i}\left( r_{1},r_{2} \right)} s_{j}w_{j}p_{j}}{\sum_{j\in\Omega_{i}\left( r_{1},r_{2} \right)} s_{j}w_{j}}$$

where:

$n_{i} = number of clusters at a distance x from cluster i, with r_{1}\leq x\leq r_{2},$

$s_{j}=number of individuals in cluster j$

$w_{j}=cluster sampling weight of cluster j$

$p_{j}=proportion susceptible in cluster j$

From this we can calculate the weighted probability of a susceptible within a distance range ${(r}_{1},r_{2})$, from all clusters:

$$\Pi\left( r_{1},r_{2} \right)=\frac{1}{\sum_{i=1}^{N} s_{i}w_{i}p_{i}}\sum_{i=1}^{N} \left\{ \frac{\sum_{j\in\Omega_{i}\left( r_{1},r_{2} \right)}^{n_{i}} s_{j}w_{j}p_{j}}{\sum_{j\in\Omega_{i}\left( r_{1},r_{2} \right)}^{n_{i}} s_{j}w_{j}}s_{i}w_{i}p_{i} \right\}$$

where:

$N = number of clusters in the survey data$

$s_{i}=number of individuals in cluster i$

$w_{i}=cluster sampling weight of cluster i$

$p_{i}=proportion susceptible in cluster i$

Finally, applying this to the original format of $\tau\left( r \right):$

$$\tau\left( r_{1},r_{2} \right)=\frac{\Pr\left( z_{i}=z_{j} \right|j\in\Omega_{i}(r_{1},r_{2}))}{\Pr\left( z_{i}=z_{j} \right|j\in\Omega_{i}(.))}$$

$$\tau\left( r_{1},r_{2} \right)=\frac{\Pi\left( r_{1},r_{2} \right)}{\Pi\left( . \right)}$$

# $\boldsymbol{\tau(r)}$ Distributions

We used an exponential function consistently characterize $\tau\left( r \right)$:

$$\tau(r) = ϴe^{-\lambda r} + \psi$$

Where $\theta$ defines the maximum probability ratio of one susceptible being in contact with another susceptible at a distance of 0 ($r=0$). $\lambda$ defines the rate of decay of the probability ratio with distance $r$. Because we are dealing with finite populations, we use $\psi$ as a rescaling value, allowing the distribution to average to 1 across the entire range of $r$. Empirical $\tau(r)$ estimates were fit to this exponential function using non-linear least squares with inverse variance weighting.

# $\boldsymbol{g}\left( \boldsymbol{r} \right)$ Distributions

We included three contact distance probability distributions ($g\left( r \right)$) in this analysis to demonstrate the use of the approach within a range of settings and to examine the sensitivity of the model to variable distributions. For *g_A_(r)* we used a published inverse cumulative probability distribution from data collected through intensive interviews and contact diaries from Guangzhou, China [3]. This population ranges from highly urban and dense to rural. Using GPS logger data from rural villages in Zambia, we constructed *g_B_(r)* using the assumption that time spent at distance $r$ from home approximated probability of contact at distance $r$[4]. This distribution represents a rural population from small villages. The final distribution, *g_C_(r)*, was constructed with cell phone data from the U.S. using *Foursquare* check-ins [5]. Though these data do not necessarily capture all contacts and contact distances for this populations, this distribution provides us with a highly dispersed $g(r)$ that may represent the personal vehicle owning and commuting populations of the U.S. Gamma distributions were fit to each distribution through mathematical optimization using the Nelder-Mead method with the *stats* R package.


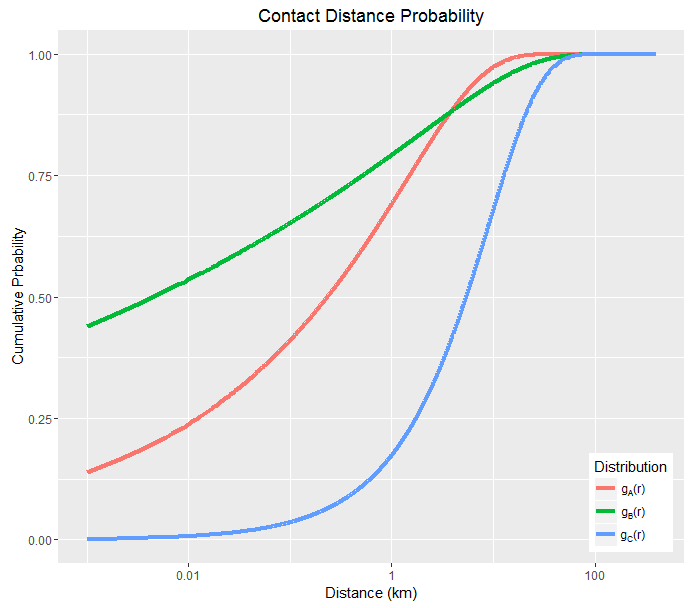


Figure S1. Contact distance probability distribution *g(r)* derived from contact diary and interview acquired contact data from China (*g_A_(r)*), GPS logger data from Zambia (*g_B_(r)*), and cell phone data from the U.S. (*g_C_(r)*) [3–5]. From these data, gamma distributions were fit to produce parameterized approximations of each distribution.

# Closed-form solution for $\boldsymbol{\phi}$

Using the exponential decay and gamma distribution forms of $\tau(r)$ and $g(r)$, we have derived a closed-form solution to the integral $\phi$. This derivation is as follows:

If $\tau\left( r \right)= \theta e^{-\lambda r}+b$ and $g\left( r \right)=f\left( r,\alpha,\beta\right)\sim gamma(\alpha,\beta)$, then:

$$\phi=\int_{0}^{\infty} \tau\left( r \right)g\left( r \right)dr$$

$$=\int_{0}^{\infty} \begin{aligned} \left( \theta e^{-\lambda r}+b \right)f\left( r, \alpha,\beta\right)dr \\ \end{aligned}$$

$$=b\int_{0}^{\infty} f\left( r,\alpha,\beta\right)dr+\theta\int_{0}^{\infty} e^{-\lambda r}\frac{\beta^{\alpha}}{\Gamma\left( \alpha\right)}r^{\alpha-1}e^{-\beta r}dr$$

$$=b+\theta\int_{0}^{\infty} \frac{\beta^{\alpha}}{\Gamma\left( \alpha\right)}r^{\alpha-1}e^{-\left( \beta+\lambda\right)r}dr$$

$$=b+\theta\frac{\beta^{\alpha}}{\left( \beta+\lambda\right)^{\alpha}}\int_{0}^{\infty} \frac{\left( \beta+\lambda\right)^{\alpha}}{\Gamma\left( \alpha\right)}r^{\alpha-1}e^{-\left( \beta+\lambda\right)r}dr$$

$$=b+\theta\frac{\beta^{\alpha}}{\left( \beta+\lambda\right)^{\alpha}}\int_{0}^{\infty} f\left( r,\alpha,\beta+\lambda\right)dr$$

$$=b+\frac{\beta^{\alpha}}{\left( \beta+\lambda\right)^{\alpha}}\theta$$

# Synthetic Populations

We validated these methods through simulation studies using spatially-explicit, synthetic populations with vaccination coverage of 85, 90, and 95% and with four defined levels of clustering of non-vaccination (*none*, *low*, *medium*, and *high*). For each vaccination level, 5 initial base populations with no susceptible clustering were generated. Each population consisted of 100,000 individuals randomly distributed within a 30x30km space. Additional sizes and densities were examined in the sensitivity analyses. Individuals were randomly selected to be non-vaccinated according to the defined level of vaccination coverage. For each level of clustering, we took base populations and stochastically clustered the vaccination status of individuals to create populations with *low*, *medium*, and *high* clustering. This heterogeneous mixing, or clustering, was performed through an iterative process of randomly swapping the vaccinated/unvaccinated statuses of randomly selected individuals until the empirical $\tau(r)$ matched the pre-defined parametric distributions (Figure S2).


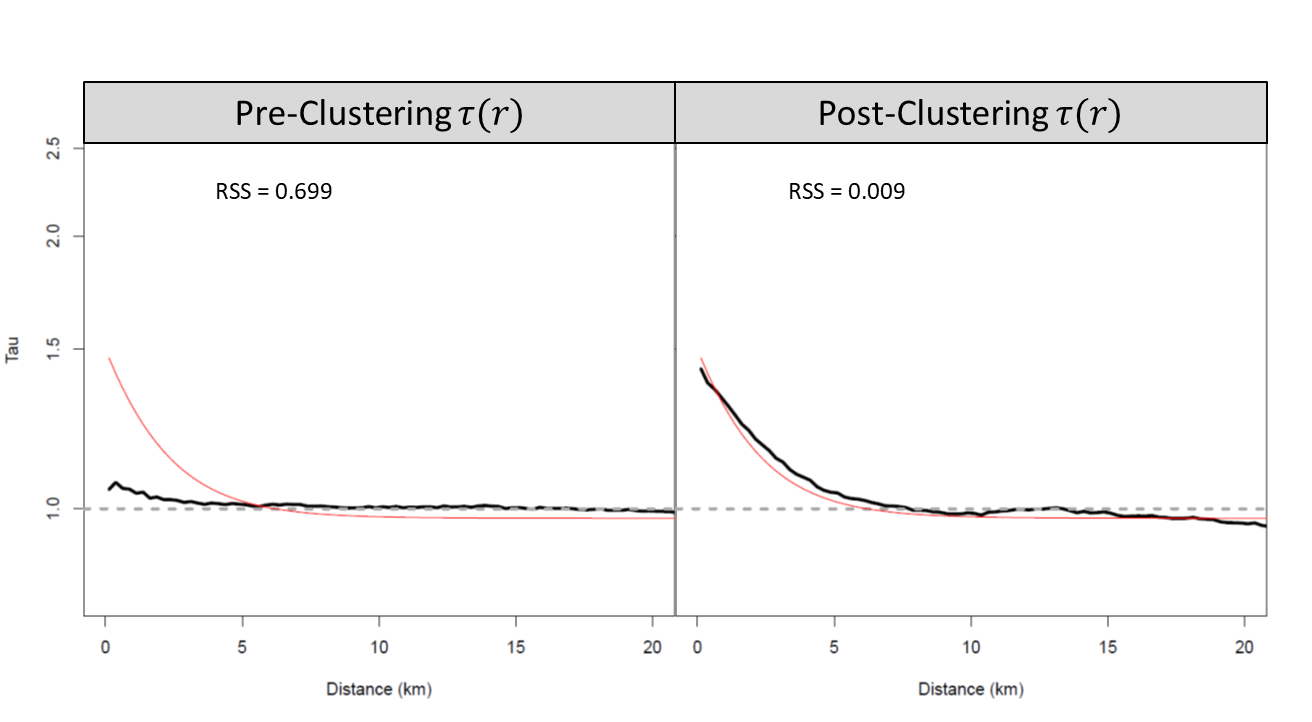


Figure S2. Example of pre-clustering $\tau\left( r \right)$ in a synthetic population and the resulting $\tau\left( r \right)$ after the vaccination clustering procedure. Red line is the pre-defined parametric $\tau(r)$ and the black line is the fitted form.

# Sensitivity Analyses

## Population Variability

Sensitivity analyses were conducted using additional synthetic populations of different sizes (N=10,000, N=50,000) and different geographic sizes (10x10km, 50x50km, etc.). These were performed to determine applicability of the methods to real populations. Our methods were found to be largely robust to both size and density. However, our methods failed in simulation to produce the expected proportions of outbreaks at lower densities, though this was expected. This resulted in localized outbreak extinctions due to rapid depletion of susceptibles in low density populations.

## Distributions

Analyses examining the impact of the *τ(d)* shape demonstrated that the $R$ estimate is relatively robust to spread ($\lambda$; Figure S3). The rate parameter of the $\tau\left( r \right)$ distribution, $\lambda_{\tau}$, also impacts the estimates produced by our approach. While $\theta,$ which is determined by the clustering level, $\lambda_{\tau}$ is determined by spread of clustering, and can represent the household-centricity of non-vaccination clustering. The $\tau(r)$ distribution narrows as $\lambda_{\tau}$ increases, and the expected $R^{*}$ produced asymptotically approaches that of the unclustered estimate.


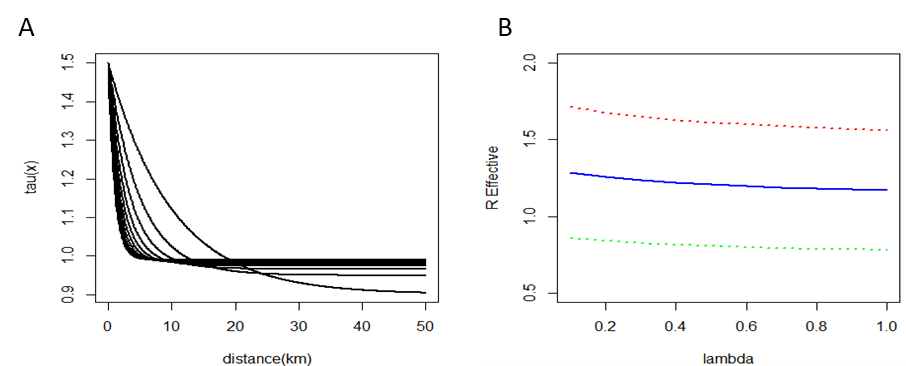


Figure S3. Effect of increasing spread of the $\tau(r)$ when $\theta$ is held constant. (A) Parametric $\tau(r)$ distributions with $\theta$=1.5. (B) Associated resulting $R^{*}$as $\lambda$ increases. Assumes a constant stable $g(r)$.

Analysis of the impact of *g(r)* shape indicated that the broader the contact distribution, the lower the resulting $R^{*}$ (Figure S4). As $g\left( r \right)$ follows a true probability distribution function, as the dispersion of the distribution increases, the peak of the distribution decreases; this is contrary to $\tau\left( r \right)$ which consist of related but independent ratios at each value of $r$. Through analyses with both the gamma and exponential distributions of $g(r)$, we see that as contact localness increases ($g(r)$ becomes steeper), departing further from homogeneous contact, $R^{*}$ increases, asymptotically approaching a clustering-dependent maximum (Figure S4).

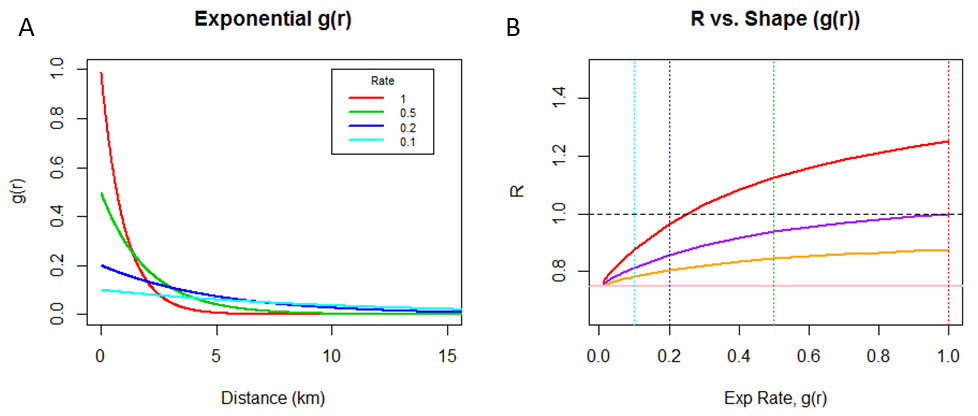


Figure S4. $g\left( r \right)$ decay rate with exponential distribution. (A) Exponential PDF forms of $g\left( r \right)$ with rate parameters 0.1, 0.2, 0.5, 1.0. (B) Exponential rate versus R for varying levels of spatial clustering.

This clustering-dependent maximum is beneficial as it limits the impact of misspecification of the exponential rate ($\lambda$) with increasing localness. For example, misspecification of $\lambda$=0.8 instead of 1.0 results in an underestimate of $R$ by 1.1% (R=1.73 vs. 1.75) at low clustering and by 3.1 % (R=2.42 vs. 2.50) at high clustering and 90% vaccination, translating to absolute $V_{c}$ underestimates of 0.06% (94.22 vs. 94.28%) and 0.13% (95.87 vs. 96.00%).


# Household-based clustering

The flexibility of our approach and the parametric distributions also allow us to experiment with real-world scenarios. For example, to verify that the model does not overestimate the risk among populations with largely household-based clustering, we can adjust the $\tau(r)$ rate parameter, $\lambda$. In such populations, we would expect a reduced risk of outbreaks compared with populations with equivalent $\theta$ values (i.e., the maximum value of $\tau(r)$), but clustering primarily outside of the household. Increasing $\lambda$, while holding θ constant, corresponds to increasing proportion of clustering contained within households, with the $\tau(r)$ distribution becomes narrower, and results in$\phi$ approaching 1, thus decreasing effect of clustering, and $R^{*}$ approaching $R$ (Figure S5).


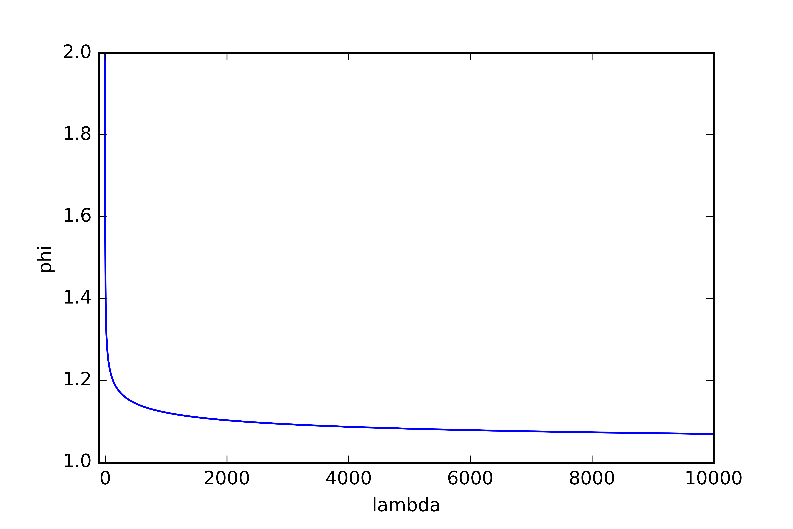


Figure S5. Impact of $\tau\left( r \right)$ rate parameter, $\lambda_{\tau}$, on the clustering coefficient, $\phi$. As $\lambda_{\tau}$ increases and the $\tau\left( r \right)$ function becomes steeper, resembling clustering centered within households, $\phi$ approaches 1 and the $R^{*}$ estimate will approach the unclustered $R$ estimate.


# Empirical $\boldsymbol{\tau}\mathbf{(}\boldsymbol{r}\mathbf{)}$ Calculations with DHS Cluster Data

To account for the characteristics of DHS surveys, and produce a final parametric $\tau(r)$ function with confidence intervals, we estimated $\tau(r)$ using a cluster-based method employed during 10,000 Monte Carlo iterations with bootstrapping. Because cluster locations are displaced for de-identification purposes, during each iteration clusters locations were jittered based on urban/rural status (urban: ≤2km, rural: ≤5km), and clusters included were bootstrapped [6]. From this jittered and bootstrapped dataset, we calculated the $\tau(r)$ function using a modified cluster-based method, which calculates $\tau\left( r \right)$ through cluster pairs rather than individual pairs, and applies the normalized sampling weights provided by DHS (Text 9.1). This calculation also employed a moving window approach for $r$, where $\tau(r)$ was calculated for overlapping $r$ windows (e.g. $r$ = 0-1km, 0.5-1.5km, 1-2km, …). The exponential decay form was then fit to the calculated empirical $\tau(r)$ from each iteration using non-linear least squares with inverse variance weighting. Means and confidence intervals of fitted parameters from each iteration were used as the final fitted $\tau(r)$ for the DHS data.

## Weighted Cluster Survey τ(r) Calculation

To calculate an accurate $\tau(r)$ using clustered survey data with sampling weights for the clusters, we use a revised calculation to account for the structure of the data and sampling weights.

The standard equation for $\tau(r)$ is:

$$\tau\left( r_{1},r_{2} \right)=\frac{\Pr\left( z_{i}=z_{j} \right|j\in\Omega_{i}(r_{1}, r_{2}))}{\Pr\left( z_{i}=z_{j} \right|j\in\Omega_{i}(.))}$$

$$\tau\left( r_{1},r_{2} \right)=\frac{\Pr\left( z_{i}=z_{j}=Z \right|j\in\Omega_{i}(r_{1}, r_{2}))}{\Pr\left( z_{j}=Z \right|j\in\Omega_{i}(r_{1}, r_{2}))}$$

_­­_ Where $Z$ is the type. In this case $Z$ = unvaccinated status.

See Salje et al. 2014 and Lessler et al. 2016 for more details.[1,2]

We designed these methods for use with both clustered survey and discrete data. As such, to use with DHS data, we aggregated the individual-level DHS data to cluster-level data, with cluster ID, geospatial coordinates, number of individuals, sampling weights, and proportion susceptible for each cluster. These methods can be directly applied to discrete data such as counties or districts.

First, we define the weighted probability of a susceptible individual within a distance range ${(r}_{1},r_{2})$ from cluster $i$:

$$\pi_{i}\left( r_{1},r_{2} \right)=\frac{\sum_{j\in\Omega_{i}(r_{1},r_{2})}^{n_{i}} s_{j}w_{j}p_{j}}{\sum_{j\in\Omega_{i}(r_{1},r_{2})}^{n_{i}} s_{j}w_{j}}$$

where:

$n_{i} = number of clusters at a distance \left( r_{1},r_{2} \right) from cluster i$

$s_{j}=number of individuals in cluster j$

$w_{j}=cluster sampling weight of cluster j$

$p_{j}=proportion susceptible in cluster j$

From this we can calculate the weighted probability of a susceptible within a distance range ${(r}_{1},r_{2})$ from another susceptible in all clusters:

$$\Pi_{\alpha}\left( r_{1},r_{2} \right)=\frac{1}{\sum_{i=1}^{N} s_{i}w_{i}p_{i}}\sum_{i=1}^{N} \left\{ \frac{\sum_{j\in\Omega_{i}\left( d_{1},d_{2} \right)}^{n_{i}} s_{j}w_{j}p_{j}}{\sum_{j\in\Omega_{i}\left( d_{1},d_{2} \right)}^{n_{i}} s_{j}w_{j}}s_{i}w_{i}p_{i} \right\}$$

where:

$N = number of clusters in the survey data$

$s_{i}=number of individuals in cluster i$

$w_{i}=cluster sampling weight of cluster i$

$p_{i}=proportion susceptible in cluster i$

It follows that we can similarly calculate the weighted probability of a susceptible within a distance range ${(r}_{1},r_{2})$ from any individual in all clusters:

$$\Pi_{\beta}\left( r_{1},r_{2} \right)=\frac{1}{\sum_{i=1}^{N} s_{i}w_{i}}\sum_{i=1}^{N} \left\{ \frac{\sum_{j\in\Omega_{i}\left( d_{1},d_{2} \right)}^{n_{i}} s_{j}w_{j}p_{j}}{\sum_{j\in\Omega_{i}\left( d_{1},d_{2} \right)}^{n_{i}} s_{j}w_{j}}s_{i}w_{i} \right\}$$

Finally, applying this to the original format of $\tau\left( r \right):$

$$\tau\left( r_{1},r_{2} \right)=\frac{\Pr\left( z_{i}=z_{j}=Z \right|j\in\Omega_{i}(r_{1}, r_{2}))}{\Pr\left( z_{j}=Z \right|j\in\Omega_{i}(r_{1}, r_{2}))}$$

$$\tau\left( r_{1},r_{2} \right)=\frac{\Pi_{\alpha}\left( r_{1},r_{2} \right)}{\Pi_{\beta}\left( r_{1},r_{2} \right)}$$

## Jittered Bootstrap Estimates

To protect the identities of surveys subjects, DHS randomly jitters the location of each cluster, with urban clusters jittered up to 2km in any direction and rural clusters jittered up to 5km. To account for this potential variability in the true locations of the clusters, we incorporated a random jitter of cluster locations into a bootstrap framework of 10,000 iterations. This improved the smoothness of the curve, particularly for distances with low numbers of cluster pairs.

## Moving Window $\tau(r)$ Estimation

To approximate a continuous $\tau(r)$ function from discrete cluster locations, we used a moving window approach, rather than a static set of discrete distance windows*.* For example, instead of using 2km discrete windows, such as 0-2km, 2-4km, 4-6km, etc. We used overlapping windows, such as 0-2km, 1-3km, 2-4km, 3-5km, 4-6km, etc. This provided a much smoother empirical estimate, particularly for distances with reduced numbers of cluster pairs.

# Technical Challenges and Limitations

## Final size comparisons

Our results demonstrate some of the technical challenges of this analysis. Dissimilarity between spatial and SIR simulation final sizes, the result of local depletion of susceptibles, makes using final size estimates to compare between models invalid (Figure S6). In this case, while probability of contact and ultimately infection of another within the SIR model is wholly dependent upon the number of susceptibles remaining, the spatial simulations employed a situation where probability of contact is dependent upon distance of susceptibles, thus when all susceptibles nearby have already been infected, the probability of a contact drops well below what it would be if numbers alone mattered.

**
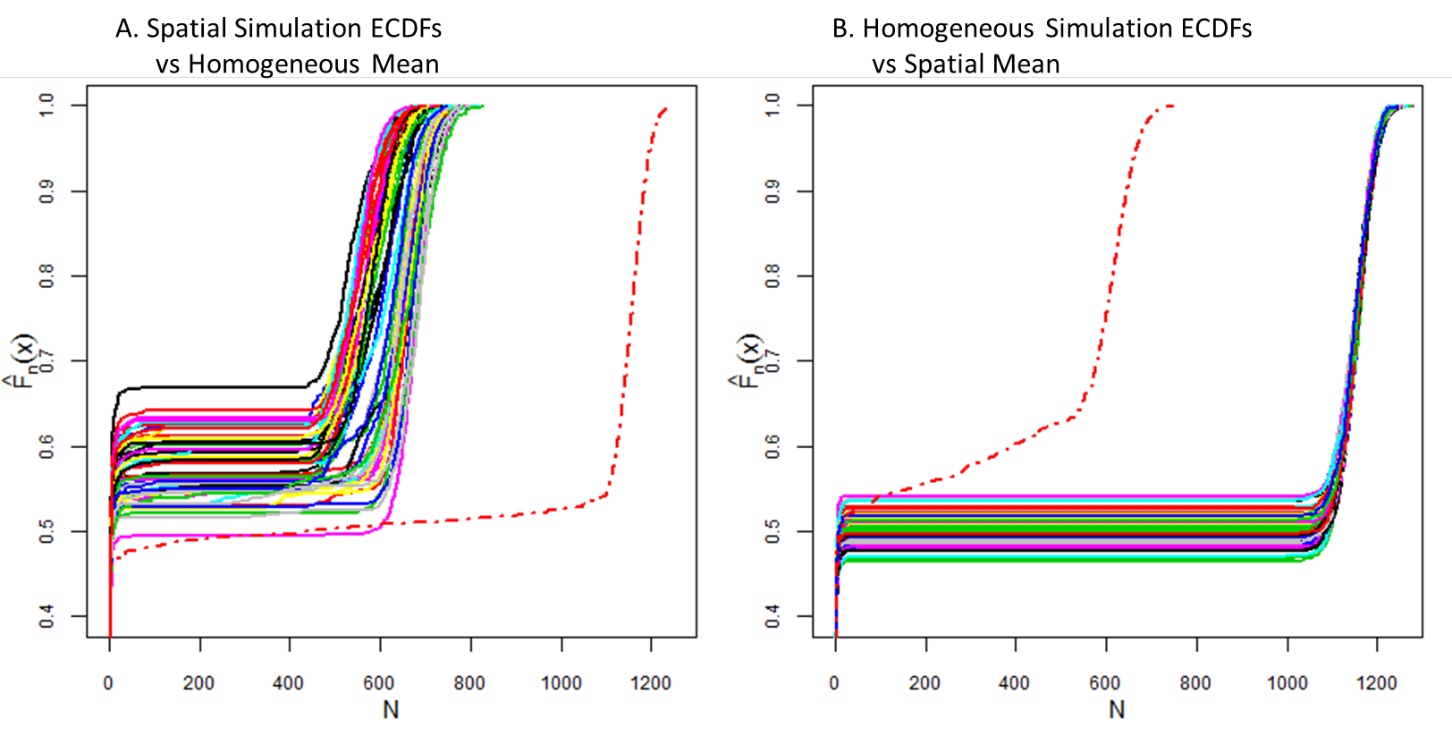
**

Figure S6. Empirical CDFs of spatial SIR simulation final sizes compared to the mean ECDF of homogeneous SIR simulations (A), and homogeneous final size ECDFs vs mean ECDF of spatial simulations. Spatial SIRs result in a mean final size around half that of homogeneous SIRs, but a similar, though slightly lower probability of outbreak.

Due to network properties, however, as $R$ increases from 1, the expected probability of an outbreak diverges in the spatial simulations from that of the SIR simulations. This divergence can be explained by the distribution of the initial individual $R$, or $R_{i}$, in the spatial populations. While $E[R_{i}]$is equal to the analytic value from Eq. 5, the distribution of $R_{i}$ is substantially right-skewed, with a large portion of the population being expected to infect several times more individuals than that of $E[R_{i}]$ (Figure S7). Furthermore, resulting from the spatial constraints of the transmission model, $R_{i}$ of subsequently infected individuals is dependent on $R_{i}$ of the infector, thus, the higher the $R_{i}$ of the infector, the higher the $R_{i}$ of the infected. This results in reduced probability of stochastic extinction and higher probability that outbreaks will propagate.­


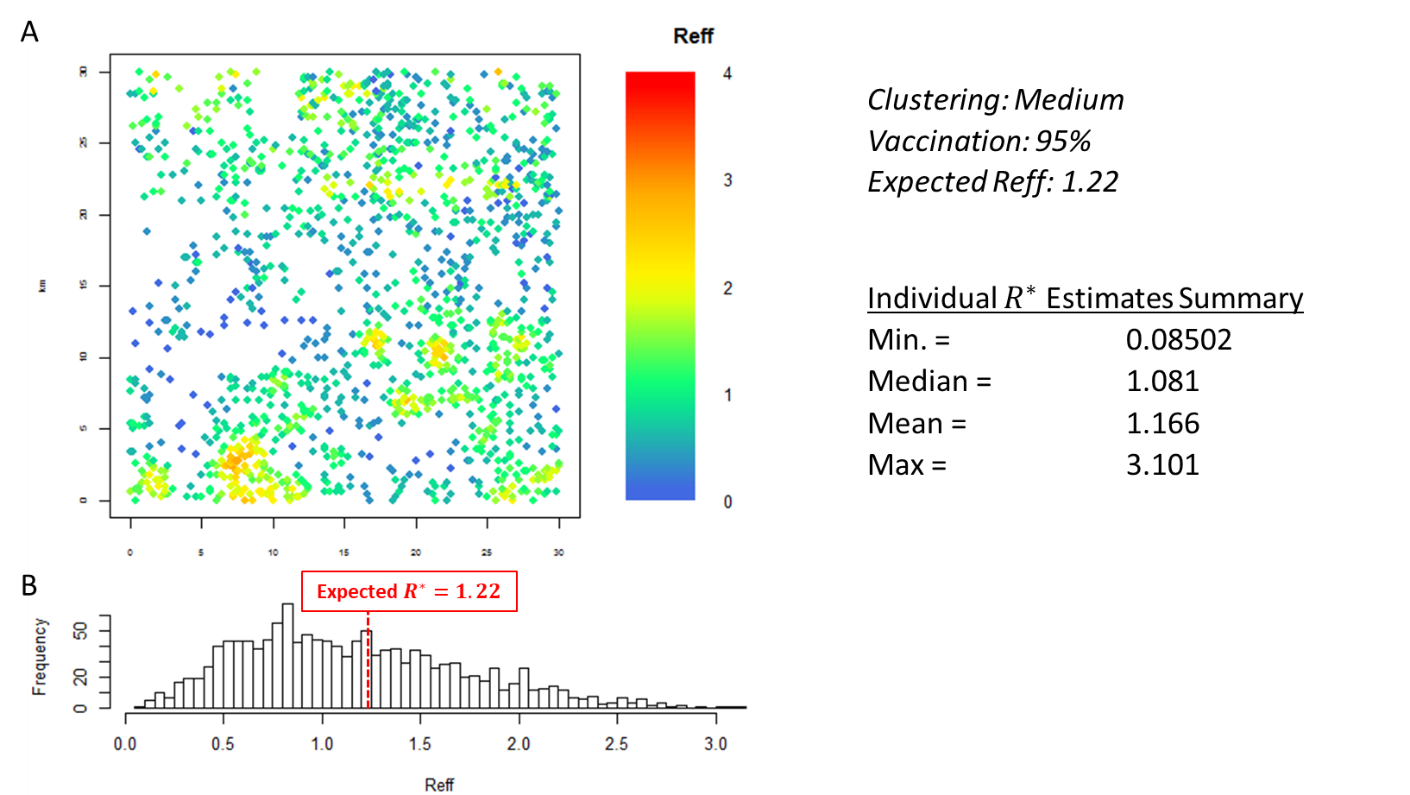


Figure S7. Individual initial $R$ for a synthetic population with *medium* clustering and 95% vaccination coverage. (A) Clustering of unvaccinated individuals in the population space (vaccinated individuals are not shown; individuals are distributed evenly in the space). Unvaccinated individuals are colored by initial $R$. (B) Distribution of initial $R$ estimates for individuals in the population.

## Vaccine efficacy and naturally-acquired immunity

Vaccine efficacy, which is believed to be around 85% for a single dose when given at nine months of age and 97% for two doses of MCV, particularly complicates use of empirical data such as DHS where we only know whether any vaccine was received or not, not the number of doses received. Additionally, low vaccine efficacy substantially increases both $R$ and the amount of vaccination needed to achieve $I_{c}$ (i.e. $V_{c}$), leading to scenarios where herd immunity is not technically achievable with single dose regimens (i.e. $V_{c}$ > 100%), especially when clustering exists (Table S3). However, vaccine efficacy is theoretically unbiased, assuming no differential cold chain or vaccine batch issues, and does not affect clustering. Thus, to account for it in our model we can simply assume $v^{*}=VE\times v$ in Equation 3.

Naturally-acquired immunity presents additional challenges to accurately estimating $R^{*}$, and if it occurs in a spatially localized manner, such as through an outbreak in a community, the nature of clustering of susceptibility may change. However, as noted above, the greatest impact of clustering, where this framework is most valuable, is when populations are close to disease elimination. In such populations, measles virus transmission is reduced or absent and immunity is largely derived from successful vaccination. Use of serological surveys could circumvent this challenge as they have the additional benefit of capturing clustering of naturally-acquired immunity [7,8].

## Contact kernel

Although specific and exact population contact data, whether simple or complex, are generally not available, we found that we can apply distributions based on population characteristics with limited error. $I_{c}^{*}$ was especially robust to minor misspecification of $g(r)$ (Figures 3 and 4), particularly among populations with highly local contact patterns, as demonstrated with $g_{A}(r)$ and $g_{B}(r)$. While derived from extremely different populations, these produced comparable $R^{*}$, $\phi$, and $I_{c}^{*}$ estimates at all levels of clustering and that differed from those of $g_{C}(r)$, where contact is much closer to homogeneous. Through identifying a set of characteristic contact distributions that capture the range of possibilities, we can likely apply this approach without need for specific contact distributions.

# Validation Challenges

To validate the results of our approach, we conducted individual-based spatially explicit simulations of transmission. Because the of contact distribution, we needed a population with both a high density and large area.

A high-density population was required because of the high probability of contacts within a short distance. As individuals are infected, they are removed from the potential pool of susceptibles. If the probability of a short contact distance is high, then the pool of susceptibles within a short distance of new and current infectious individuals becomes depleted, reducing probability of additional infections. Furthermore, if susceptible individuals are clustered, as we are proposing, this further complicates the depletion of susceptibles, as the distance to the next cluster might be relatively high, thus the probability of a transmission to that cluster is low. In this case, as soon as the cluster is depleted, the outbreak dies out, potentially below the threshold of an outbreak. However, by increasing the density of the population overall, the numbers of individuals, and thus chance for longer distance transmission increases.

Additionally, as the contact distance distribution, $g(r)$, continues to infer some probability of transmission at higher distance, we need to make the population area large enough to not artificially truncate $g(r)$ and bias the distance of contacts toward shorter distances.

Complicating these validation procedures, the higher the density and area of the population, the larger the population size, and the larger the number of susceptibles. For our validations, we are introducing infection by randomly selecting a single susceptible individual to be infectious, and simulated the transmission that results. Furthermore, once selected, the process of infectious transmission is stochastic, thus 100 simulations with the same initial infectious individual could all produce different results. When we have base populations of 500,000 with 90% vaccination, thus 50,000 susceptible individuals, running enough simulations to simply capture an iteration where each susceptible is the initial infectious individuals is computationally intensive. Doing this for multiple populations for each of the 12 vaccination coverage/clustering level combinations requires high computational capacity.

# Additional Supplemental Tables and Figures

Table S1. Non-vaccination clustering adjustment factor estimates for the three contact distance distributions and four levels of clustering of non-vaccination.

| **Clustering  Level** | ***Clustering Adjustment Factor (*φ*)*** | | |
| --- | --- | --- | --- |
|  | Read et al. 2014, *g_A_(r)* | Rural Zambia, *g_B_(r)* | Noulas et al. 2012, *g_C_(r)* |
| None | 1.00 | 1.00 | 1.00 |
| Low | 1.18 | 1.20 | 1.06 |
| Medium | 1.36 | 1.40 | 1.12 |
| High | 1.71 | 1.80 | 1.24 |

Table S2. Non-vaccination clustering-adjusted effective reproductive number estimates for 85, 90, and 95% effective vaccination coverage at the three contact distance distributions and four levels of non-vaccination clustering. Assumes $R_{0}$=15.

| **Effective Vaccination Coverage** | **Clustering** | ***R****† | | |
| --- | --- | --- | --- | --- |
|  |  | ***g_A_(r)*** | ***g_B_(r)*** | ***g_C_(r)*** |
| **95%** | *None* | 0.75 | 0.75 | 0.75 |
|  | *Low* | 0.89 | 0.90 | 0.80 |
|  | *Medium* | 1.03 | 1.06 | 0.85 |
|  | *High* | 1.31 | 1.36 | 0.94 |
| **90%** | *None* | 1.50 | 1.50 | 1.50 |
|  | *Low* | 1.78 | 1.81 | 1.60 |
|  | *Medium* | 2.06 | 2.11 | 1.69 |
|  | *High* | 2.61 | 2.72 | 1.89 |
| **85%** | *None* | 2.25 | 2.25 | 2.25 |
|  | *Low* | 2.67 | 2.71 | 2.39 |
|  | *Medium* | 3.08 | 3.17 | 2.54 |
|  | *High* | 3.92 | 4.08 | 2.83 |
|  |  |  |  |  |
| † *Assumes R­_0_=15 and* $\lambda$*=0.5* | | | | |

Table S3. Non-vaccination clustering-adjusted critical vaccination thresholds at *none*, *low*, *medium*, and *high* clustering with vaccine efficacy (VE) of 100% and 95%, with $\lambda$=0.5.

| **Vaccine Efficacy** | **Clustering Level** | ***V_c_*** | | |
| --- | --- | --- | --- | --- |
|  |  | **Read et al. 2014, *g_A_(r)*** | **Rural Zambia, *g_B_(r)*** | **Noulas et al. 2012, *g_C_(r)*** |
| **100%** | *None* | 93.3% | 93.3% | 93.3% |
|  | *Low* | 94.3% | 94.4% | 93.7% |
|  | *Medium* | 95.1% | 95.2% | 94.0% |
|  | *High* | 96.1% | 96.3% | 94.6% |
| **95%** | *None* | 98.2% | 98.2% | 98.2% |
|  | *Low* | 99.3% | 99.4% | 98.6% |
|  | *Medium* | >100% | >100% | 99.0% |
|  | *High* | >100% | >100% | 99.6% |

Table S4. Non-vaccination clustering-adjusted critical vaccination thresholds at *none*, *low*, *medium*, and *high* clustering with vaccine efficacy (VE) of 100% and 95%, with $\lambda$=0.5.

| **Vaccination Coverage** | **Clustering** | **R***‡ | **Probability of Outbreak**† | |
| --- | --- | --- | --- | --- |
|  |  |  | **Spatial Simulations,** *mean (95% CI)* | **SIR Simulations,** *mean (95% CI)* |
| **95%** | None | 0.75 | 0 (0-0.01) | 0 (0-0) |
|  | Low | 0.89 | 0.01 (0.00-0.02) | 0.01 (0.01-0.01) |
|  | Medium | 1.03 | 0.07 (0.06-0.08) | 0.07 (0.07-0.08) |
|  | High | 1.24 | 0.23 (0.22-0.24) | 0.23 (0.22-0.24) |
|  |  |  |  |  |
| **90%** | None | 1.45 | 0.32 (0.28-0.36) | 0.36 (0.34-0.37) |
|  | Low | 1.78 | 0.43 (0.38-0.47) | 0.48 (0.46-0.50) |
|  | Medium | 2.01 | 0.47 (0.44-0.50) | 0.52 (0.50-0.54) |
|  | High | 2.31 | 0.52 (0.48-0.57) | 0.59 (0.56-0.62) |
|  |  |  |  |  |
| **85%** | None | 2.24 | 0.58 (0.51-0.66) | 0.61 (0.58-0.63) |
|  | Low | 2.61 | 0.64 (0.52-0.76) | 0.65 (0.63-0.68) |
|  | Medium | 3.08 | 0.70 (0.58-0.82) | 0.73 (0.69-0.76) |
|  | High | 3.92 | 0.75 (0.61-0.89) | 0.79 (0.76-0.83) |
| *† Outbreak is defined by* $\geq$*5% of the susceptible individuals becoming infected. Assumes introduction happens at equal rates randomly among susceptibles.*  *‡* $R^{*}$ *calculated analytically using the empirical g(r) from spatial simulations and defined* $\tau(r)$ *distributions.*  *¥ Relative risk of outbreak occurring in spatial simulations, where “None” is the reference for each vaccination coverage level. Reference is no clustering. RR for SIR simulation results.* | | | | |

Table S5. Incidence and estimated effective reproductive numbers for Tanzania from 1998 to 2014.

| **Year** | **Routine Vaccination** | **Incidence (per 100,000)** | **R, unadjusted**** | **R, adjusted***** | ***V_c_*, adj.** |
| --- | --- | --- | --- | --- | --- |
| 1998 | 72% | 31.0 | 4.2 | 5.2 | 94.6% |
| 1999* | 72% | 17.8 | 4.2 | 5.3 | 94.7% |
| 2000 | 78% | 43.1 | 3.3 | 4.3 | 94.8% |
| 2001 | 86% | 34.0 | 2.1 | 2.8 | 94.9% |
| 2002 | 89% | 14.3 | 1.6 | 2.2 | 95.0% |
| 2003 | 97% | 4.5 | 0.5 | 0.6 | 95.1% |
| 2004 | 94% | 3.7 | 0.9 | 1.3 | 95.2% |
| 2005 | 91% | 0.1 | 1.3 | 1.9 | 95.3% |
| 2006 | 93% | 5.9 | 1.0 | 1.5 | 95.4% |
| 2007 | 90% | 18.6 | 1.5 | 2.2 | 95.5% |
| 2008 | 88% | 8.0 | 1.8 | 2.7 | 95.6% |
| 2009 | 91% | 3.6 | 1.3 | 2.1 | 95.6% |
| 2010* | 92% | 0.4 | 1.2 | 1.9 | 95.7% |
| 2011 | 93% | 3.4 | 1.0 | 1.7 | 95.8% |
| 2012 | 97% | 3.4 | 0.5 | 0.7 | 95.9% |
| 2013 | 99% | 0.4 | 0.2 | 0.2 | 95.9% |
| 2014 | 99% | 0.2 | 0.2 | 0.3 | 96.0% |
| ** Years for which DHS data is available; from these the τ(r) functions for Tanzania were calculated to estimate clustering of susceptible individuals.  ** Unadjusted effective reproductive number; calculated as X=X­_0_(1-v). *** Adjusted effective reproductive number; calculated as X=X­_0_(1-v)φ, where φ is assumed to change linearly between 1999 and 2010.* | | | | | |

Figure S8. (A) Probability of an outbreak with increasing clustering ($\phi$) and effective reproductive number ($R$). This assumes an introduction has occurred and an outbreak is defined as 5% of susceptible individuals becoming infected. (B) Probability ratio (PrR) of an outbreak with increasing clustering ($\phi$) compared with homogeneous ($\phi$=1).


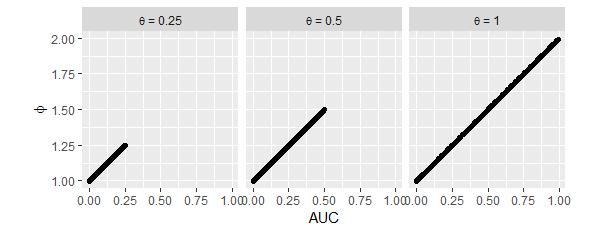


Figure S9. Correlation between $\phi$ and AUC of $1-G(r)$ versus $\tau(r)$ curves. All three levels of clustering ($\theta$=0.25, 0.5, 1.0) have perfect correlation with AUC (Pearson’s *r*=1.0). For each combination of $\tau(r)$ and $g(r)$, $\phi$ = AUC + 1.


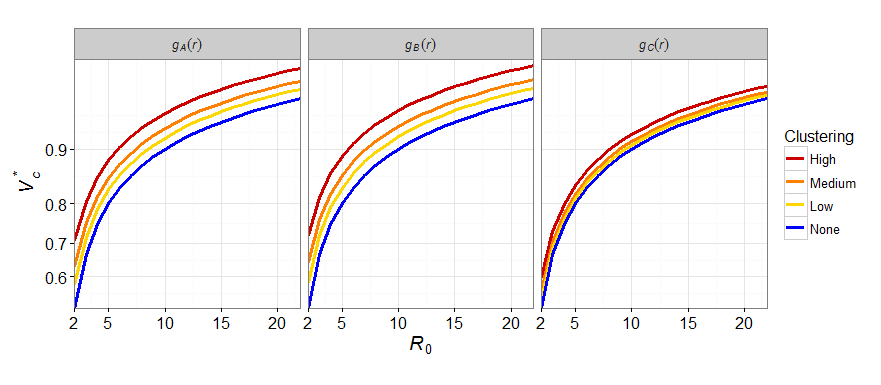

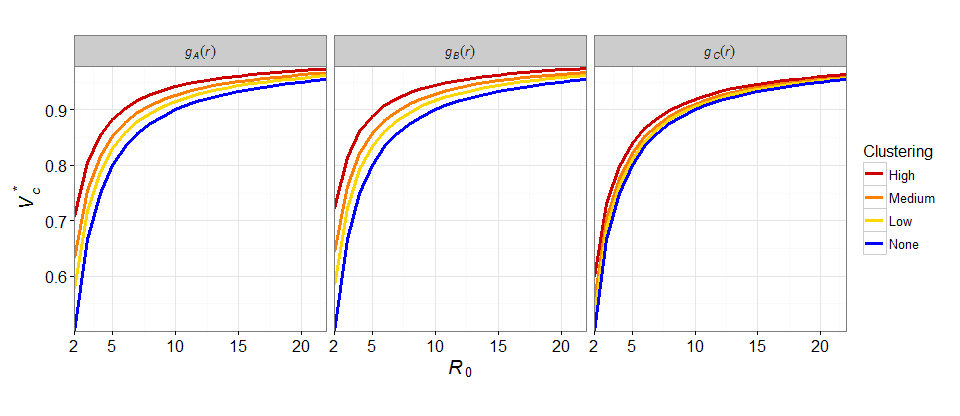


A

B

Figure S10. The association between $R_{0}$ and $V_{c}^{*}$ at the four defined levels of clustering and three $g\left( r \right)$ distributions. As clustering increases, the required vaccination coverage to maintain R=1 increases. At low $R_{0}$, this increase due to clustering is much greater than at high $R_{0}$. (A) Linear $V_{c}^{*}$ scale. (B) Logarithmic $V_{c}^{*}$ scale.


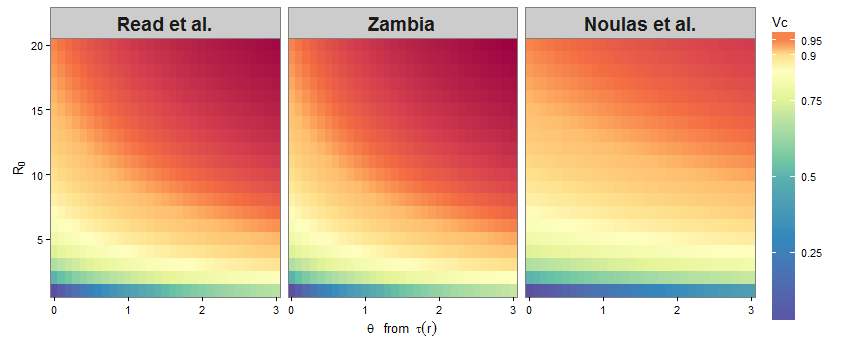


Figure S11. Association between $R_{0}$, clustering maximum( $\theta$ parameter of $\tau(r)$), and the critical vaccination threshold ($V_{c}$).


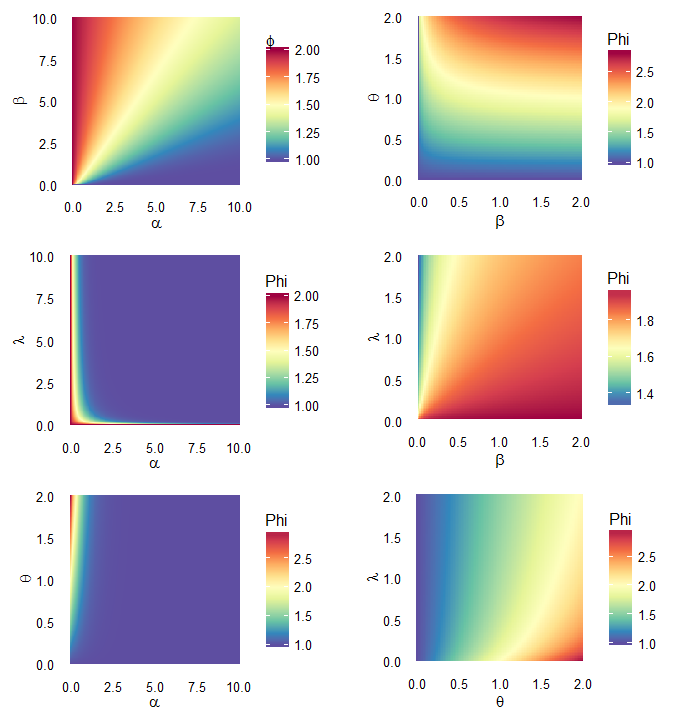


Figure S12. Sensitivity analyses of the varied impact of $\tau\left( x \right)$ parameters ($\theta$ and $\lambda$) and $g\left( x \right)$ parameters (shape and scale) on the resulting $R_{eff}$ estimate. $\tau\left( x \right)$ follows an exponential distribution characterized as $\tau\left( x \right)=\theta e^{-\lambda x}+\psi$, while $g\left( x \right)$ follows a standard gamma distribution parameterized by shape and scale parameters. Regardless of the $g\left( x \right)$ parameters, as $\theta$ increases, $R_{eff}$ increases (A and B).


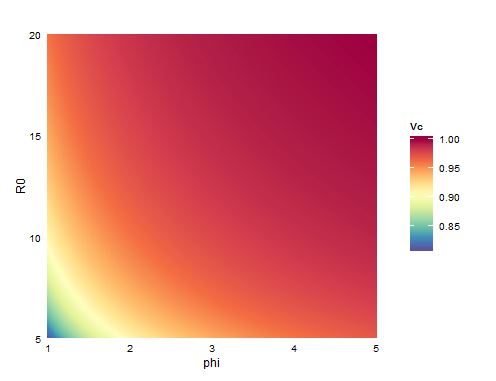


Figure S13. Relationship between $R_{0}$, $\phi$, and $V_{c}$.

Figure S14. Analytic $R^{*}$ estimates for measles assuming $R{}_{0}$=15 and using contact distributions derived from Read et al. 2014 (*g_A_(r)*) and Noulas et al. 2012 (*g_C_(r)*) [3,5]. As clustering increases, R increases; however, this difference decreases as vaccination coverage increases.

References

1. Salje H *et al.* 2012 Revealing the microscale spatial signature of dengue transmission and immunity in an urban population. *Proc. Natl. Acad. Sci.* **109**, 9535–9538. (doi:10.1073/pnas.1120621109)

2. Lessler J, Salje H, Grabowski MK, Cummings DAT. 2016 Measuring Spatial Dependence for Infectious Disease Epidemiology. *PloS One* **11**, e0155249. (doi:10.1371/journal.pone.0155249)

3. Read JM, Lessler J, Riley S, Wang S, Tan LJ, Kwok KO, Guan Y, Jiang CQ, Cummings DAT. 2014 Social mixing patterns in rural and urban areas of southern China. *Proc. Biol. Sci.* **281**, 20140268. (doi:10.1098/rspb.2014.0268)

4. Searle KM, Lubinda J, Hamapumbu H, Shields TM, Curriero FC, Smith DL, Thuma PE, Moss WJ. 2017 Characterizing and quantifying human movement patterns using GPS data loggers in an area approaching malaria elimination in rural southern Zambia. *R. Soc. Open Sci.* **4**, 170046. (doi:10.1098/rsos.170046)

5. Noulas A, Scellato S, Lambiotte R, Pontil M, Mascolo C. 2012 A Tale of Many Cities: Universal Patterns in Human Urban Mobility. *PLoS ONE* **7**, e37027. (doi:10.1371/journal.pone.0037027)

6. In press. The DHS Program - Quality information to plan, monitor and improve population, health, and nutrition programs. See http://www.dhsprogram.com/ (accessed on 27 June 2016).

7. Lessler J, Metcalf CJE, Cutts FT, Grenfell BT. 2016 Impact on Epidemic Measles of Vaccination Campaigns Triggered by Disease Outbreaks or Serosurveys: A Modeling Study. *PLOS Med.* **13**, e1002144. (doi:10.1371/journal.pmed.1002144)

8. Metcalf CJE, Farrar J, Cutts FT, Basta NE, Graham AL, Lessler J, Ferguson NM, Burke DS, Grenfell BT. 2016 Use of serological surveys to generate key insights into the changing global landscape of infectious disease. *The Lancet* **388**, 728–730. (doi:10.1016/S0140-6736(16)30164-7)
